# Perceived stress and lower back pain amongst nurses during the SARS-CoV-2, across hospitals in Durban, South Africa

**DOI:** 10.4102/curationis.v48i1.2698

**Published:** 2025-05-06

**Authors:** Laralyn L. Naidoo, Jed L. Davidson

**Affiliations:** 1 Department of Biokinetics, Exercise and Leisure Sciences, Faculty of Health Sciences, University of KwaZulu-Natal, Durban, South Africa

**Keywords:** lower back pain, stress, COVID-19, nurses, health

## Abstract

**Background:**

The coronavirus disease 2019 (COVID-19) pandemic strained the healthcare sector and workers. Nurses experienced stress and burnout because of strain in resources, limited staff and exposure risk. Resultant lower back pain was prevalent. Nurses were poorly equipped to manage these conditions physically and psychologically.

**Objectives:**

The study aims to investigate lower back pain and stress levels during COVID-19. It also aims to provide data describing any association between both.

**Method:**

Pain and stress were determined using questionnaires. Quantitative, descriptive design and convenient sampling were used. The Chi-square goodness-of-fit-test tests significant Back Pain Functional Scale (BPFS) responses (12 daily activities lower back pain) and determines the relationship between pain and stress before and during COVID-19 related to the current time. Chi-square test of independence is used on cross-tabulations to determine the relationship between stress and lower back pain. Fisher’s exact test was used for conditions not met. The Binomial test was used for the significance check of yes/no response to medication use. A questionnaire was provided on exercise levels and provision by workplace.

**Results:**

Higher pain and stress were noted during versus prior to COVID-19. Back Pain Functional Scale showed no difficulty performing the majority of activities. A small percentage used medication, showing no significant change. Majority did not perform exercise for reducing lower back pain prior to or during COVID-19. Exercise intervention was not provided by workplaces for the majority, during or prior to COVID-19.

**Conclusion:**

Lower back pain and perceived stress levels increased during the COVID-19 pandemic. Provision of lower back pain education and exercise intervention in preventing and managing lower back pain in hospital nurses was needed. This study adds to the stress and lower back pain knowledge base in South Africa.

**Contribution:**

The findings assist in understanding the effects of Covid-19 on stress and lower back pain in nurses, linkage of the two, and possible interventions to reduce these effects using knowledge enhancement and prescribed exercise interventions.

## Introduction

Coronavirus disease 2019 (COVID-19) is a severely infective and easily transmissible disease, caused by severe acute respiratory syndrome coronavirus 2 (SARS-CoV-2). It was first discovered in Wuhan, Hubei Province, China, in late December 2019 and spread rapidly across the world (Sethi et al. 2020). It was declared a global pandemic on 11 March 2020 by the World Health Organization (WHO). The virus mutated into several variants and strains, which caused repeated and severe illness worldwide. Coronavirus disease 2019 can be transferred via direct contact (exposure to infected respiratory droplets and via others) or indirect contact (airborne transmission) (Lotfi, Hamblin & Rezaei 2020). The most common form of transmission is exposure to infected respiratory droplets and contamination of touch surfaces (Cascella et al. 2023).

In a South African context, the first case of COVID-19 in the country was detected on 05 March 2020 in KwaZulu-Natal. Community transmission occurred rapidly; a national state of disaster was thereafter declared (Moonasar et al. 2021). Yang and Shaman (2022) showed that between March 2020 and August 2020, the number of COVID-19 cases peaked. Severe acute respiratory syndrome coronavirus 2 resulted in many waves of the pandemic with a rapid spread in various locations. The second wave, which posed a greater threat, peaked in January 2021, with the third wave peaking in June 2021. Between 05 March 2020 and 27 March 2021, a total of 1 545 431 SARS-CoV-2 cases and 227 932 COVID-19 hospital admissions were reported in South Africa (Jassat et al. 2021).

This unprecedented health emergency of course hugely impacted the national health system. However, it must be noted that before the COVID-19 pandemic, the South African healthcare system was already under strain. This was because of multiple burdens in diseases, a lack of health human resources, poor governance and management and unequal distribution of resources among provinces and between the public and private healthcare sectors (Engelbrecht, Heunis & Kigozi 2021). Nurses are the first point of contact in hospital medical protocols, and nurses were experiencing stressful conditions prior to the breakout of the COVID-19 pandemic (Engelbrecht et al. 2021). Oftentimes, they are required to carry out tasks in the absence of availability of other healthcare workers. This often causes an increase in the workload of nurses, contributing to the development of lower back pain. The daily tasks of nurses require them to lift patients, as well as transport them or move equipment (Kasa et al. 2020). This causes overloading on the back when performed incorrectly. A load applied to the back that is sudden in nature and unexpected can result in lower back pain, such as lifting of an object with improper technique and lack of awareness of the mass of the object (Van der Burg et al. 2000). Multiple factors cause the inability of the individuals back structures to manage the load, such as decreased muscle activity in relation to the load on the back. Additionally, when the trunk extensor muscle fibres have limited activation, this results in extensive flexion of the trunk caused by gravity (Dixit 2017). There is an increase in the active muscles being lengthened (eccentric), which can lead to muscles becoming disrupted or causing injury to the muscle tissue, compared to when the muscle is shortened (concentric) (Van der Burg et al. 2000).

During a pandemic and resultant crisis, nurses are tasked with more roles and responsibilities, including more taxing physical undertakings. Therefore, nurses must be adequately equipped with knowledge and specific skills to effectively manage the crisis, negating any stress that may develop as much as possible. Nurses were involved in screening issues, collaborating treatments of patients, as well as taking care of patients with disease. This involved clinical treatment, appropriate decontaminating, isolating measures, effective communication, triaging, the provision of support that is psychological and necessary palliative care (Thobaity & Alshammarib 2020). Nurses across the globe experienced limitations in knowledge, lack of leisure and recovery hours, with accompanying psychological and family burdens (Firouzkouhi et al. 2022). Comparing South Africa to the rest of the world, an urgent organisation of healthcare workers was required. Swift development of scientific and public health information was needed, which nurses had to adapt to, and carry out recommendations in strained healthcare facilities. Despite actioned measures, South Africa had the highest number of cases of COVID-19 and deaths in Africa (Mokhele et al. 2022).

Apart from periods of specific and unanticipated impact, stress in general occurs in everyday life and can be caused by changes to the environment or political, social and economic variations (Metreveli & Japaridze 2022). It involves the body responding to a demanding situation by attempting to maintain balance. Factors or events leading to the stress are referred to as stressors. There are several responses to stressors, such as when the body undergoes changes physically or physiologically, impact from the surrounding environment and events of life or behaviours (Sharma 2018). Stress affects all the systems that involve bodily functions, namely, the cardiovascular, respiratory, endocrine, gastrointestinal, nervous, muscular and reproductive systems. Stress has both a direct and indirect effect on health by altering the autonomic and neuroendocrine responses and causing behavioural health changes. The body possesses survival adaptations to both the internal and external environment known as homeostasis (O’Connor et al. 2021).

The symptoms of stress result in the release of a hormone known as cortisol via the nervous and endocrine system. Cortisol is a stress hormone released via the bloodstream, which promotes the catabolic processes in the body. Stimulation of the spine may cause temporary interruptions to the body’s homeostatic processes in individuals presenting with lower back pain. Physiological stress can occur as a result of chronic lower back pain, thereby causing the body to release increased levels of cortisol, in an uncontrolled manner. Individuals who present with chronic pain and increased stress levels have an increase in cortisol levels compared to those who are healthy (Seyed & Mohamed 2021). Altered endocrine function can be a predisposing factor for the recurrence of pain that becomes chronic in nature. This results in an increased release of proinflammatory neurotransmitters such as norepinephrine, resulting in peripheral muscle nociceptor-sensitisation. Consequently, the muscles locally become tensed and stabilise without a return to a relaxed state, thus resulting in stiffness and pain (Buscemi 2025). Physical factors result in musculoskeletal pain, and in addition, psychological factors such as work-related risk factors may also result in musculoskeletal pain. Work-related psychological factors include stress, repetitive tasks, increased workloads and time pressures. Karlibel et al. (2020) showed in a study conducted to determine the relationship between burn-out and lower back pain, that lower back pain was common among healthcare workers who experienced burdens in a physical and psychological nature during the pandemic.

Homeostasis occurs to ensure optimal internal bodily functions for the various physiological states (O’Connor et al. 2021). The inability of the body to resolve a stressful situation results in it consuming all or dysfunctional amounts of its resources. This occurs by secreting stress hormones continuously or abnormally, providing energy to resolve the situation. Physical changes include muscular tension, joint pain and body pains (Sharma 2018). The musculoskeletal system is affected by stress and causes the muscles to tense up forming a guarding mechanism against further pain and possible injury (Chu et al. 2022).

With regard to impact on the body, pain can be described as the body’s vitally important, biological protective response with an adaptive function, and is classified as acute or chronic in nature (Raffaeli & Arnaudo 2017). Chronic pain exists for longer than 3 months where the cause is not definitive or of an unknown origin (Bonezzi et al. 2020). A transition may occur from acute pain to chronic pain. Chronic pain can even arise without an actual injury. This transition causes the central nervous system Central Nervous System (CNS) to undergo changes in function and morphology, which is a result of acute persistent nociceptive (i.e., pain) stimulation (Mosabbir 2022).

In terms of specific areas of the body that are most often affected by chronic pain, stress and dysfunction, lower back pain is described as located in the back, beginning at the lower end of the ribcage to the buttocks and may or may not include discomfort in the lower limbs (Chen et al. 2021). Lower back pain is described as mechanical (pain that originates from soft tissue and spinal areas such as discs, joints and vertebrae) or non-specific (pain that occurs with or without a known cause) (Casiano et al. 2023). The healthcare system is often impacted by lower back pain because of its high prevalence and disability associated with it. Lower back pain decreases productivity and increases absenteeism of individuals. Lower back pain hinders basic activities of daily living, which causes limitations to functional movements. It also impacts life generally, causing changes to physical and mental well-being, and affects relationships socially (Husky et al. 2018). Because of the complexity of chronic back pain, there is no ‘cure’ for it, with alleviating causative factors displaying no guarantee in complete relief. Pain neuroscience education (PNE) is therefore vital in improving and understanding pain, therefore reducing the threat pain poses to individuals (Mosabbir 2022).

One of the most important positive behavioural activities to maintain health, avoid pain and reduce stress is of course exercise. Exercise is physical activity that involves a plan, structure and repetition to improve and maintain levels of physical fitness (Stennett, De Souza & Norris 2020). Exercise plays an overall role in the general health and well-being of individuals, having both mental and physical benefits to the body and is important in managing chronic health conditions, one of which is chronic lower back pain. Exercise assists in increasing functional capacity to perform activities of daily living more efficiently (Dreisinger 2014). It can play a role in preventing lower back pain, secondarily assisting in reducing pain intensity and reoccurrence and a prevention strategy for the development of pain in individuals that also do not experience pain (Ijabadeniyi & Fasae 2022). Exercise therapy for lower back pain can have a moderate effect in decreasing pain and disabling concerns and is a cost-effective method in pain reduction compared to other strategies. Various types of exercises are used for treating chronic lower back pain such as general aerobic training, strengthening exercises and more holistic modalities such as pilates and yoga (Grooten et al. 2021).

Instead of treatment, another strategy is prevention, and prevention of lower back pain can also be achieved via the use of educational and exercise interventions (Zhang, Wan & Wang 2014). Health education can positively assist nurses in various settings in preventing or reducing lower back pain. Health education and conventional care can lead to a better quality of life, and pain and disability will be decreased. Education should comprise an overview of the spinal column and back anatomy, accurate posture maintenance, use of body mechanics and information provided on suitable exercises for maintaining lower back health. Ideal weight and lifestyle education are important, and information on the causes of lower back pain will assist in preventing the onset of lower back pain and/or the management of its symptoms (Güneş & Ayaz-Alkaya 2021).

Coronavirus disease 2019 posed a negative effect on the physical and mental health of medical professionals and a large proportion of the nursing population in particular were affected. Common mental health detriments included depression, anxiety and insomnia. Approximately 50.4% of medical professionals experienced depressive episodes, while anxiety because of stressful situations occurred in approximately 90% of healthcare workers. Common stress factors included situations arising as a result of the pandemic, attributed to negative emotions such as feelings of danger, being uncertain, frustration and anger (Tomaszewska et al. 2022). The outcomes of an investigation to determine the stressors of nurses during the COVID-19 pandemic, indicated that the highest assessed stressor was the fear of becoming infected, followed by death, patients dying and work overload (Lorente, Vera & Peiró 2020).

### Aim and objectives

The study aimed to investigate perceived stress levels and lower back pain during the COVID-19 peak in public and private hospital nurses in Durban, South Africa. The objectives were to determine pre- and post-COVID-19 levels of the occurrence of lower back pain and perceived stress in the sample of nurses and investigate any association. Additionally, the study aimed to determine the extent and influence of pain medication and exercise on lower back pain.

## Research methods and design

### Study design

The study used a quantitative design that was descriptive in nature, utilised a questionnaire and provided a summary and explanation of the data collected and the condition investigated.

### Setting

Participants were selected from public and private hospitals across Durban, South Africa. The setting included a variety of nurses from various age groups and backgrounds.

### Study population and sampling strategy

The inclusion criteria were nurses with the presence of chronic or intermittent lower back pain between March 2020 and March 2021, providing services during the pandemic peak period. Any nurses with lower back pain because of an accident, deformity, injury to the spine, congenital complications, pathological, student or temporarily employed nurses were excluded from the study. The intended sample size was 113 participants, calculated using Stata v17, with the final sample size of 86, because of accessibility limitations. Convenience sampling, a non-probability sampling, was used to increase access to participants because of nurses’ working hours. Participants were recruited via correspondence through hospital management. Thereafter, nurses were approached individually in each ward, following approval from the nursing unit manager. Sample selection aimed to represent the target population and eliminate any confounding factors. The researcher made efforts to ensure the participants sampled matched the population to maintain consistency and accuracy by informing participants of the inclusion and exclusion criteria required in the research before participation.

### Data collection

The Back Pain Functional Scale (BPFS), a practical evaluation method, was used to determine the functional ability of individuals with lower back pain. This self-report includes 12 short actions used to determine the level of function of individuals with lower back pain. This tool was selected because of its validity and reliability, with sensitivity to clinical setting variations. The scale has a detectable change of 22.2% with a standard error of measure of 6.5% at a 95% confidence interval. The test–retest reliability is high, with intra-class correlation coefficient of 0.88 at a confidence interval of 77% (Stratford et al. 2000). Thereafter, participants were required to indicate the level of stress and lower back pain via the use of three comparative indications (higher, lower or the same), prior to COVID-19 compared to during COVID-19 (March 2020–March 2021), via the use of a subsequent section of the questionnaire, comprising four related questions. This provided information on the comparison of lower back pain and stress levels before COVID-19, and during COVID-19, to determine the effect this period had on the stress level and lower back pain. The comparative indications prior to and during COVID-19 were used to indicate the severity of stress levels and lower back pain. The use of medication specifically for lower back pain was then determined prior to COVID-19 and/or during COVID-19. These questions were used to determine the severity of the lower back pain and the extent of this method of management of the pain. Participants then answered questions that provided information regarding their current exercise levels, exercise levels prior to COVID-19 and during COVID-19 (March 2020–March 2021) and if they were provided with any physical activity interventions and/or carried out any interventions for their lower back pain prevention or management.

### Data analysis

The data were entered into electronic spreadsheets, where all the responses were displayed individually for all participants for all the questions. Descriptive statistics including means and standard deviations were used to analyse data initially. The Chi-square goodness-of-fit-test is a univariate test that is used on a variable that is categorical. In this study, it was used to test whether the options of the responses that were selected were significant in their occurrence. This was used for the BPFS to determine whether any of the response options were selected significantly more than others and to determine the relationship between pain and stress before and during COVID-19 in relation to current time. The Chi-square test of independence was used on cross-tabulations to establish whether a significant relationship exists between the two variables represented in cross-tabulation. When conditions were not met, Fisher’s exact test is used. The Binomial test was used to assess whether a significant proportion of respondents selected one of a possible two responses – yes or no to the use of medication.

### Ethical considerations

In line with these ethical responsibilities, the study proposal was first presented at a postgraduate research presentation session. Thereafter, the study plan was submitted to the UKZN Human Social Sciences Research Ethics Committee (HSSREC) for ethical approval prior to the commencement of data collection. Ethical clearance was then obtained via a granted clearance letter from HSSREC (HSSREC/00004575/2022). Thereafter, approval from the public and private sector was obtained. The public sector clearance was obtained from the Department of Health (DoH) (National Health Research Database reference no.: KZ_202209_031). The private sector approval was obtained from the research operations committee (approval number: UNIV-2023-0021).

All data collected were stored securely with only the researcher having access to the completed questionnaires and electronic data. As per UKZN’s research policy, data are stored at the premises and will be destroyed after 5 years. Only the researcher and supervisor have access to this data. Before commencement of data collection, the hospital management was first notified and upon the approval, data collection then proceeded. The participant was first explained the purpose of the study and that participation was voluntary. Upon agreement of participation, the consent form was signed prior to answering the questionnaires. The participant was made fully aware of the purpose of the study, how the study was to be carried out, the advantages of the study on the nursing profession, their voluntary participation with a right to withdraw at any given time and the steps taken by the researcher to ensure confidentiality and anonymity. Upon data collection, the researcher visited the hospital wards with the unit manager consent. The researcher ensured that patients in that ward were not neglected during the research by selecting participants that were not busy during that time, and participants were allowed sufficient time, considering any patient requirements during filling out the questionnaire.

Confidentiality and anonymity were ensured by the researcher, who further emphasised that the participants were not to include their names when completing the questionnaire. The information sheet emphasised that the data collected would not be linked to any participant. Participants were reminded that management would not have access to individual data linking to the participant, thereby ensuring that the research will not potentially impact their jobs negatively. Each participant was also made aware of this in the information sheet.

## Results

### Demographic profile

A total of 86 nurses participated in the study, ranging between 24 and 63 years of age, with 81 (94.2%) female participants and five (5.8%) male participants. This aligns to data provided by WHO in 2019, stating that in South Africa, male nurses form 9.1% of the population, whereas female nurses form 90.9% of the population. The public sector included 13 (15.1%) participants with 73 (84.9%) in the private sector. The mean age of the sample was 39.16, with a standard deviation of 8.584.

### Lower back pain and stress in nurses prior to coronavirus disease 2019 and during coronavirus disease 2019, compared to current time

**FIGURE 1:**
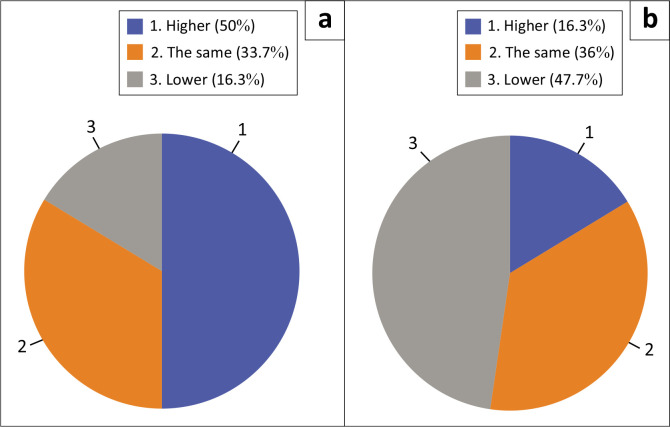
Lower back pain: (a) during the coronavirus disease 2019 pandemic and (b) prior to the coronavirus disease 2019 pandemic.

**FIGURE 2:**
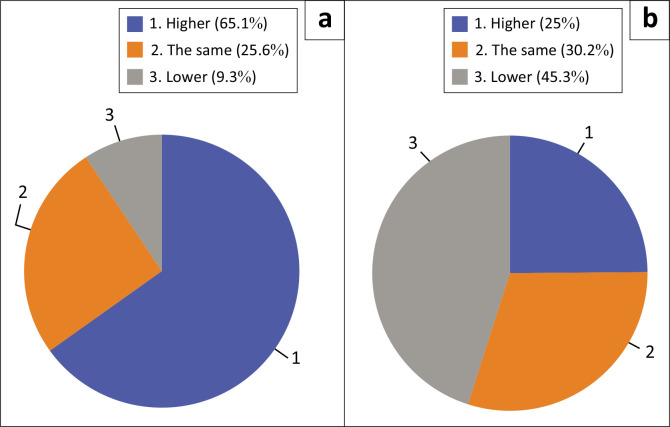
Stress: (a) during the coronavirus disease 2019 pandemic and (b) prior to the coronavirus disease 2019 pandemic.

Participants responded to the questions about their stress and lower back pain, indicating if it was higher, lower or the same. Participants whose lower back pain was higher *during* COVID-19 (50.0%) were significantly *greater* than *prior* to COVID-19 (16.3%), *p* < 0.001. The proportion of participants whose stress levels were higher *during* COVID-19 (65.1%) was significantly *greater* than *prior* to COVID-19 (24.4%), *p* < 0.001 (Figure 1 and Figure 2).

### Back pain functional scale

The BPFS was used to assess back pain prevalence and measure and evaluate the functional ability of individuals with lower back pain. Overall, majority indicated that they were able to perform activities with no difficulty or little difficulty (Table 1), indicating that generally lower back pain did not affect the nurses’ functionality. However, variances in responses were found across different activities.

**TABLE 1:** Responses as frequency of the back pain functional scale.

Activity	Responses as frequency (%)						χ^2^	*df*	*p*
	Unable to perform – 0	Extreme difficulty – 1	Quite a bit of difficulty – 2	Moderate difficulty – 3	A little difficulty – 4	No difficulty – 5			
Usual work housework or school activities	1.2	14.0	14.0	14.0	18.6	38.4	38.05	5	< 0.001
Usual hobbies recreational/ sporting activities	17.4	8.1	16.3	11.6	17.4	29.1	13.07	5	0.023
Performing heavy activities around your home	5.8	14.0	16.3	17.4	18.6	27.9	13.21	5	0.021
Bending or stooping	2.3	9.3	15.1	16.3	17.4	39.5	40.56	5	< 0.001
Putting your shoes or socks	1.2	9.3	7.0	16.3	12.8	53.5	90.79	5	< 0.001
Lifting a box of groceries from the floor	2.3	12.8	14.0	11.6	17.4	41.9	45.86	5	< 0.001
Sleeping	2.3	10.5	5.8	19.8	**18.6**	43.0	55.21	5	< 0.001
Standing for 1 h	1.2	8.1	16.3	10.5	16.3	47.7	67.77	5	< 0.001
Walking 1 mile (1.6 km)	5.8	14.0	11.6	5.8	20.9	41.9	47.54	5	< 0.001
Going up or down 2 flights of stairs (about 20 steps)	4.7	11.6	16.3	10.5	20.9	36.0	31.07	5	< 0.001
Sitting for 1 h	1.2	3.5	9.3	12.8	15.1	58.1	113.81	5	< 0.001
Driving for 1 h	2.3	3.5	7.0	16.3	15.1	55.8	103.63	5	< 0.001

χ^2^, Chi-square; *df*, degrees of freedom.

The Chi-square goodness-of-fit test was used to determine the significance of the response where the participant was required to answer based on the period of the pandemic (March 2020–March 2021). Various statistically significant outcomes were found in the data related to levels of difficulty performing activities. There was little difficulty performing eight of the activities – any of their usual work housework or school activities (*18.6%,* χ^2^ *38.05, p* ≤ *0.001*), their usual hobbies recreational or sporting activities (*17.4%,* χ^2^ *13.07, p = 0.023*), performing heavy activities around their home (*18.6%,* χ^2^ *13.21, p = 0.021*), bending or stooping (*17.4%,* χ^2^ *40.56, p* ≤ *0.001*), lifting a box of groceries from the floor (*17.4%,* χ^2^ *45.86, p* ≤ *0.001*), sleeping (*18.6%,* χ^2^ *55.21, p* ≤ *0.001*), walking 1 mile (1.6 km) (*20.9%,* χ^2^ *47.54, p* ≤ *0.001*) and going up or down two flights of stairs (about 20 steps) (*20.9%,* χ^2^ *31.07, p* ≤ *0.001*). Activities reported as *moderately difficult* included performing heavy activities around their home (*17.4%,* χ^2^ *13.21, p = 0.021*) and sleeping (*19.8%,* χ^2^ *55.21, p* ≤ *0.001*). Participants *unable to perform* their usual hobbies recreational/sporting activities because of their lower back pain comprised *17.4%* of the sample (χ^2^ 13.07, *p* = 0.023).

### Perceived stress levels and lower back pain in the public and private sectors

*During* the COVID-19 pandemic, 35 nurses (*47.9%*) in the private sector experienced higher *lower back pain* compared to the public sector, where 8 45 nurses (*61.5%*) experienced higher *lower back pain. Prior* to the COVID-19 pandemic, 10 nurses (*13.7%*) in the private sector experienced higher *lower back pain* compared to the public sector, showing 4 (*30.8%*) experienced higher *lower back pain*.

*During* the COVID-19 pandemic, 45 nurses (*61.6%*) in the private sector experienced higher *stress* levels compared to the public sector where 11 (*84.6%*) experienced higher *stress* levels. *Prior* to the COVID-19 pandemic, 15 nurses (*20.5%*) in the private sector experienced higher *stress* levels as compared to the public sector where 6 (*46.2%*) experienced higher *stress* levels.

### Correlation between stress and lower back pain

Ordinal scale was used and Spearman’s correlation was applied. A moderate positive correlation between pain and stress during COVID-19 was found (rho = 0.335, *p* = 0.002). There was also a high positive correlation between pain and stress prior to COVID-19 (rho = 0.623, *p* < 0.001). Higher levels of stress are associated with higher levels of pain.

### Lower back pain levels prior to coronavirus disease 2019 and during coronavirus disease 2019

Majority of the sample of nurses had an increase in lower back pain during COVID-19 and with lower levels of lower back pain prior to COVID-19 (Table 2), indicating a relationship between pain prior to and during COVID-19, when compared to current pain (χ^2^ [4] = 61.351, *p* < 0.001). When comparing pain to the current time, 12 (85.5%) nurses experienced *higher* pain both prior to and during COVID-19, whereas 28 (83.9%) nurses experienced the *same* pain both prior to and during COVID-19. Twelve (29.3%) nurses experienced *decreased* lower back pain during COVID-19, whereas 28 (68.3%) nurses experienced *higher* pain during COVID-19.

**TABLE 2:** Percentage of reported lower back pain levels prior to the coronavirus disease 2019 and during the coronavirus disease 2019.

Pain	Percentage during COVID-19			Chi-square	*p*
	Higher	The same	Lower		
**Prior to COVID-19**					
Higher	85.7	14.3	0.0	11.560	< 0.009
The same	9.7	83.9	5.0	-	-
Lower	68.3	2.4	29.3	-	-

COVID-19, coronavirus disease 2019.

### Perceived stress levels prior to coronavirus disease 2019 and during coronavirus disease 2019

The largest proportion of the sample experienced higher stress levels during COVID-19 and lower levels of stress prior to COVID-19 (Table 3).

**TABLE 3:** Percentage of perceived stress levels prior to coronavirus disease 2019 and during coronavirus disease 2019.

Stress	Percentage during COVID-19			Fisher’s exact	*p*
	Higher	The same	Lower		
**Prior to COVID-19**					
Higher	85.7	0.0	14.3	57.390	0.001
The same	19.2	80.8	0.0	-	-
Lower	84.6	2.6	12.8	-	-

COVID-19, coronavirus disease 2019.

Participants were required to indicate, compared to present stress levels, if their stress was higher, the same or lower prior to the COVID-19 pandemic and during the peak (March 2020–March 2021) of the COVID-19 pandemic. Participants were aware that the comparison was between three time periods ‘prior, during and currently’, before answering. Data were analysed using the Fischer’s exact test. When comparing stress to the present time, 3 (14.3%) participants experienced lower stress prior to the pandemic, and 33 (84.6%) experienced higher stress during the pandemic.

### Pain medication

Results indicated that 21 (24.4%) participants took medication for their lower back pain during the pandemic. The binomial test was applied to determine whether a significant proportion of participants responded yes or no. Additionally, 22 (26%) reported to be currently taking medication for lower back pain. A significant section of the sample, 12 nurses (14%) did indicate that pain medication was required during the pandemic.

### Relationship between pain and medication

The Pearson’s Chi-square was used to analyse data related to lower back pain and medication usage. Results indicate that 10 participants (37.2%) who suffered from higher pain prior to COVID-19 (compared to current pain) took medication for lower back pain during the COVID-19 pandemic. Results also show that 12 participants (42.9%) who suffered from higher pain prior to COVID-19 (compared to current pain) took medication for lower back pain prior to COVID-19. Medication was required for their lower back pain because of its imposed limitation in carrying out their jobs and because of the pain present after completing a shift.

### Exercise engagement

A set of questions were used to determine whether participants engaged in exercise generally, to determine exercise levels before and during the COVID-19 pandemic and if specifically, to reduce lower back pain. Additionally, questions were posed to establish whether their workplace provided strategies to utilise exercise or physical activities to reduce lower back pain before and during the COVID-19 pandemic (Table 4).

**TABLE 4:** Exercise levels in nurses.

Exercise	%	
	Yes	No
1. Do you currently exercise? (general)	47.7	52.3
2. Did you exercise prior to the COVID-19 pandemic (March 2020 – March 2021)?	45.3	54.7
3. Did you start performing exercises prior to the Covid-19 pandemic (March 2020 – March 2021) to reduce your lower back pain? If yes, what type/s of exercise? Type of exercise:	23.3	76.7
4. Did you start performing exercises during the COVID-19 pandemic (March 2020 - March 2021) to reduce your lower back pain? If yes, what type/s of exercise? Type of exercise:	20.9	79.1
5. Did your workplace provide you with any strategies to utilize exercise to reduce lower back pain prior to the COVID-19 pandemic (March 2020 - March 2021)? If yes, what type/s of exercise? Type of exercise:	14.0	86.0
6. Did your workplace provide you with any strategies to utilize exercise to reduce lower back pain during to the COVID-19 pandemic (March 2020 - March 2021)? If yes, what type/s of exercise? Type of exercise:	**10.5**	89.5

### Exercise levels in nurses

The responses indicate an absence of current time exercise in a slight majority of the participants, 45 nurses (52.3%), whereas the rest of the sample of nurses did report to be engaging in exercise, 41 (47.7%). Majority of the sample, 47 (54.7%), indicated that they did not perform exercise prior to the COVID-19 pandemic, whereas the rest of the population did exercise prior, 39 (45.3%). Exercising did not have much change when comparing exercise levels prior to versus during COVID-19. The highest percentage of the sample did not perform exercise to reduce their lower back pain prior to the COVID-19 pandemic, 66 (76.7%). The rest of the nurses formed a much lower percentage, which did perform exercises prior to COVID-19 to reduce their lower back pain, 20 (23.3%). Similar number was found for during COVID-19; the majority of the sample indicated that they did not perform exercises to reduce their lower back pain, 68 nurses (79.1%), with a small percentage in comparison performing exercises to reduce their lower back pain during the height of the pandemic, 18 (20.9%).

Most of the participants in the study (74 nurses, 86%) indicated that their workplace did not provide any physical activity strategies, with a small percentage indicating that their workplace did implement these pain reduction approaches (12, 14.0%). Participants then indicated if their workplace provided strategies to utilise exercise to reduce their lower back pain *during* the COVID-19 pandemic, with the highest percentage (77 nurses, 89.5%) of responses indicating no, while a small percentage indicated that their workplace did (9, 10.5%). This important result indicates that exercise intervention was not provided by their workplaces generally, and a larger number of participants indicated that exercise interventions were not provided *during* the pandemic.

### Types of exercises

Six participants indicated that they started performing exercises *prior to* the COVID-19 pandemic (March 2020–March 2021) specifically to reduce their lower back pain. The responses included: two participants performed back stretching, one reported aerobics and line dancing, one indicated lunges, meditation, Pilates and squats, one performed cardio and one indicated running as a response.

The same six participants indicated they performed exercises *during* the COVID-19 pandemic (March 2020–March 2021) specifically to reduce their lower back pain. The exercises included: one participant who performed sciatic exercises and stretches, one back stretching, one included walking and stretching, one aerobics and line dancing, another participant Pilates and cardio by another participant. The physical activity or exercises provided by the workplace interventions to reduce lower back pain during and prior to the COVID-19 pandemic, indicated by a small percentage of participants, included stretching and techniques on lifting a patient.

Minimal exercise was done for the participant’s lower back pain. From the exercises that the nurses did engage in, a variety in the types was noted. There were no clearly defined patterns for the reduction of lower back pain.

## Discussion

### Key findings

Following the evaluation of stress and lower back pain in this sample of South African nurses and influencing factors, the study indicated that there was an increase in perceived stress and an increase in lower back pain during the peak of the COVID-19 pandemic. Most nurses with lower back pain did not engage in exercise before or during COVID-19 to reduce or prevent pain, and few had intervention provided to them from employers.

### Discussion of key findings

With regard to the impact of pain on the work and personal functioning of the participants, BPFS results indicated that while overall pain impact was not prominent, a small portion of the participants were unable to perform their usual hobbies recreational/sporting activities because of their lower back pain. Similarly, a study undertaken by Bozic et al. (2022) with 512 nurses showed that because of the presence of lower back pain, 68.40% of nurses had a reduction in leisure activities. Sayılan et al. (2021) also showed when lower back pain in nurses was investigated, that out of a sample of 234 nurses, most of the activities affected because of the presence of lower back pain were basic activities of daily living and social activities, 56.5%. Participants who found difficulties with weight lifting, standing, housework and social activities comprised 13.8%.

When looking at lower back pain prior to and during COVID-19, 28 nurses experienced higher lower back pain during COVID-19 and decreased lower back pain prior to COVID-19. Research data compiled by Ozen and Cakmak (2021) to determine the prevalence of chronic lower back pain in healthcare workers during the COVID-19 pandemic found that from a sample of 170 participants of multiple healthcare workers, chronic lower back pain was most prevalent in the nursing population. A total of 67.9% of the sample of healthcare workers experienced pain prior to the COVID-19 pandemic. The number of participants whose lower back pain was increased during the COVID-19 pandemic formed the majority of the sample in this study, 96 participants (57.5%). Despite the presence and history of lower back pain before the onset of the COVID-19 pandemic, the majority of the lower back pain complaints increased during the pandemic. Similarly, a study by Kasa et al. (2020) displayed the prevalence of lower back pain in nurses working in a clinical setting in Africa in a meta-analysis and used a sample size of 6110. The time of this study was from 2000 to 2018. The lowest prevalence of lower back pain was 44.1%, and the highest prevalence was 82.7%, found in a study conducted in Nigeria. When the subgroups were analysed, it was noted that the highest prevalence was found in the West African region, 68.46%, followed by North Africa, 67.95%. These two African regions had a higher prevalence rate as compared to South Africa, 59%. In agreement with these results, a study undertaken in Jeddah, Saudi Arabia, displayed the prevalence of lower back pain in nursing professionals, showing the incident rate of lower back pain using 60 subjects was 61.7%. The study concluded that the nurses working in that hospital had a high prevalence of lower back pain (Gaowgzeh 2019).

Results indicated that 84.6% of the sample of nurses in the current study experienced higher stress during the pandemic. Investigations into stress levels of nurses during the pandemic have revealed similar results. A study carried out by Tomaszewska et al. (2022) utilising 118 nurses working with COVID-19 patients reported 47.5% (*n* = 56) of respondents indicated that they felt stress at work with every duty, and 43.2% (*n* = 51) indicated that they sometimes felt stress at work. The conclusion was that a higher number of the respondents indicated that they had stress at work during the COVID-19 pandemic. Zakeri et al. (2021) investigated anxiety, stress and depression during the COVID-19 first wave. These three factors increased significantly during the first wave of the COVID-19 pandemic compared to before the COVID-19 breakout (*p* < 0.05). The study was conducted prior to (April 2020 – July 2020) and during (April 2020 – July 2020) the first wave of the COVID-19 pandemic (Zakeri et al. 2021). In agreement, Çınar and other researchers investigated perceived stress and factors related to the COVID-19 pandemic. Similarly, it was found that out of a sample of 169 nurses, 44.6% experienced elevated stress, which was affected by their working conditions (Çınar et al. 2021).

There is a large discrepancy between the sample size of the public and private sector, with private having majority; however, results show that the public sector experienced higher stress levels and lower back pain compared to the private sector. This is also shown when Tsegaw et al. (2022) investigated stress across public and private hospitals with 304 nurses, an overall stress percentage value across both public and private hospitals was stated as 48.40%. The public sector (51.60%) had higher stress levels compared to the private sector (46.40%) (Tsegaw et al. 2022). Results indicate that 85.7% of nurses in the current study experienced higher lower back pain during and prior to the COVID-19 pandemic; however, 68.3% nurses experienced increased lower back pain during COVID-19 and decreased lower back pain prior to COVID-19. Similarly, a comparative study carried out in Iran in 2017 using 203 nurses involved in critical care, aimed to determine the occupational stress between the public and private sector nurses. The overall mean (SD) score of occupational stress experienced by these nurses in public hospitals was greater than that experienced in private hospitals, 3.65 (0.77) vs. 3.18 (0.94). This indicated that occupational stress was higher in the public sector as compared to the private sector (Chegini 2019).

A study by Vinstrup, Jakobsen and Andersen (2020) conducted across 19 hospitals in Denmark, which aimed to determine perceived stress and lower back pain among healthcare workers, also shows similar results. The results of the nursing population in particular indicated that an increase in stress levels was associated with an increased chance of lower back pain developing. Choobineh et al. (2021) also highlight the prevalence of lower back pain found to be 69.9%, using a sample of 495 nurses. This study investigated the association between job stress dimensions and the prevalence of lower back pain. Based on the job stress dimensions, the only dimension that had a significant (*p* < 0.05) association with lower back pain was psychological demands, which was linked to high levels of stress, therefore indicating another association between stress and lower back pain in the literature.

When looking at the last sections of the questionnaire related to *Medication and exercise for lower back pain*, more nurses began taking medication for their lower back pain during (21%) the pandemic compared to prior (14%) the pandemic. Similarly, a study carried out by Kanakkarthodi et al. (2022), where lower back pain was analysed in the nursing population, showed that out of 182 nurses that experienced lower back pain, 43 nurses had taken medication for their pain and 110 nurses did not receive treatment. Likewise, Al-Samawi and Awad (2015) reported in a study analysing the incidence of lower back pain in a sample of 80 nurses, that 14.3% do not do anything for pain relief, whereas 5.7% makes use of a pharmacologic method for relief, 37.1% used non-pharmacologic methods for relief and 42.9% used both. The use of pain medication for lower back pain provides relief differently for all individuals. Research completed by Lalitha and Singh (2022) showed that out of 50 nurses, the effectiveness of medication for their lower back pain varied. Forty two per cent experienced full relief, 26% received moderate relief and 14% experienced no change in pain with medication. Gündüz and Sayılan (2021) also showed the usage of medication amongst the nursing population for their lower back pain comprised 53.8% (*n* = 126) of the 234 nurses that participated in the study.

Kore et al. (2021) analysed lower back pain and associated factors among nurses working in hospitals in Ethiopia and found that nurses who performed physical exercise regularly were 73% less likely to experience lower back pain compared to those who did not perform regular physical exercises. Shieh et al. (2016) analysed an increased lower back pain risk in nurses. Out of the 567 who had lower back pain, 481 (85.7%) did not perform exercises. Ijabadeniyi and Fasae (2022) also showed the prevalence of lower back pain in 200 nurses; results indicated that 118 (59%) did not engage in regular exercise, which is also a predisposing factor of lower back pain. Fiter, Werdhani and Wahyuni (2018) sought to determine the effect of back-exercise on the level of lower back pain in hospital nurses. Back pain was analysed before and after the intervention. From the 20 participants, none of participants performed regular exercise. There was a significant difference in lower back pain in nurses after the back-exercise intervention (pelvic tilting, double knee chest, bridging, prone plank and side-lying abduction movement) was carried out. Pain decreased and functionality increased in the nurses, indicating that back strengthening exercises reduce lower back pain.

Similarly, a study using 50 nurses in the hospital setting with mechanical lower back pain carried out on intervention to determine the effect of back strengthening in reducing mechanical lower back pain. The exercises of the intervention included pelvic tilt, sit up, knee to chest stretch, double knee stretch and straight leg raise stretch. A pre-test and post-pain assessment were done by the researchers. Back strengthening interventions have shown that back exercises are effective in reducing lower back pain (Lalitha & Singh 2022).

In a study conducted to determine the knowledge, attitude and perception of lower back pain exercise among nurses in a public hospital setting, out of 191 nurses, the knowledge of nurses was identified to be 45.3%, perception was 54.5% and attitude was 28.3%. Knowledge was categorised (76% – 100%) as good knowledge, 51% – 75 % moderate knowledge and 0 – 50% slight knowledge. The study concluded that the knowledge of nurses about their lower back pain was limited. Therefore, indicating that if more knowledge and education are provided, the prevalence of lower back pain can be reduced (Sultana et al. 2022). In agreement with this, a study carried out on the prevalence of musculoskeletal lower back pain among nurses was analysed. The study aimed to evaluate scientific findings available. After critically analysing eight studies, it was found that despite the development of the 21st century, lower back pain prevalence among nurses was high. This was because of the lack of standardised effective preventative measures of occupational safety globally. The literature reviewed showed that the occurrence of lower back pain was a result of the conditions nurses worked in, being unaware of the occupational risks and decreased focus on preventative and safe manual handling education (Gilchrist & Pokorná 2020).

### Strengths and limitations

A standardised questionnaire was utilised, with the researcher present to answer and clarify any questions that arose during data collection. The study fills gaps in existing South African and global knowledge by providing more information on the association between stress and lower back pain. Results provide more indication of how stress and lower back pain are related, creating awareness among the population and employers, thereby possibly increasing productivity, decreasing absenteeism and improving efficiency. The study outcome may enhance prioritisation of psychosocial care in private and public health sectors. Psychosocial care will assist in managing stressful situations better thereby improving the nurse’s work quality and work ability mentally. Statistical tools were used to analyse data, correlating and concluding objectives, with the assistance of a statistician. Limitations included peak/busy working hours because of nurses being frontline workers, as they were engaged with basic routine tasks of the position. Finding an appropriate time to explain the study and questionnaire was challenging in some cases because of changes in shifts, lunch times/breaks or the requirements of patients arising during the data collection. Recall impact on the data is possible, related to time frame of presentation of stress, lower back pain and answering of questions, as the study was specifically for the time period in which COVID-19 was at the peak (having the highest infection rate), March 2020–March 2021. Questionnaire fatigue may have been a factor, as nurses were required to read through information sheets, informed consent and then questionnaires. The risk of dishonesty of participants is a possible factor, as they may not have indicated their accurate stress or lower back pain. Anxiety/stress may have affected data accuracy and related recall, because of the questionnaire being set during the peak of the pandemic, when stressful events had occurred.

### Implications or recommendations

There are limited studies on the association of lower back pain and stress specifically during the peak of the COVID-19 pandemic. Future research to include larger sample size overall is recommended to show data and gather information for more of the nursing population. A larger sample size in the public sector, which will assist in obtaining more data from both the public and private sector, would be useful for future comparison. More research sites can be included in subsequent studies to display a variety of participants from different hospitals or settings. More emphasis can be placed on nurses including exercise as a preventative or treatment measure for lower back pain, as opposed to the use of medication, which will also prevent lengthy time periods off work. The provision of education and strategies to minimise lower back pain by more workplaces can be advised. Workplaces can provide psychosocial support for nurses to manage their stress levels and provide education on management and prevention of stress holistically. The above mentioned will assist in appropriately managing stress and lower back pain if another pandemic arises. This will improve the ability to manage the working conditions, decrease lower back pain and stress levels.

## Conclusion

Nurses in the private and public sector experienced an increased level of lower back pain and stress during the COVID-19 pandemic peak (March 2020–March 2021). Lower back pain and stress were found to be higher in the public compared to the private sector. Some nurses also began taking medication to reduce their lower back pain during the pandemic. The majority of nurses in the study did not make use of exercise as a pain management or preventative strategy. Results also showed that the majority of hospitals that nurses worked at did not provide strategies to reduce or manage their lower back pain.
